# Genetic analyses in a bonobo (*Pan paniscus*) with arrhythmogenic right ventricular cardiomyopathy

**DOI:** 10.1038/s41598-018-22334-5

**Published:** 2018-03-12

**Authors:** Patrícia B. S. Celestino-Soper, Ty C. Lynnes, Lili Zhang, Karen Ouyang, Samuel Wann, Victoria L. Clyde, Matteo Vatta

**Affiliations:** 1 Department of Medical and Molecular Genetics, Indiana University School of Medicine, Indianapolis, IN 46202 USA; 2 Milwaukee County Zoo, Milwaukee, WI 53226 USA; 3 Krannert Institute of Cardiology, Division of Cardiology, Department of Medicine, Indiana University School of Medicine, Indianapolis, IN 46202 USA

## Abstract

Arrhythmogenic right ventricular cardiomyopathy (ARVC) is a disorder that may lead to sudden death and can affect humans and other primates. In 2012, the alpha male bonobo of the Milwaukee County Zoo died suddenly and histologic evaluation found features of ARVC. This study sought to discover a possible genetic cause for ARVC in this individual. We sequenced our subject’s DNA to search for deleterious variants in genes involved in cardiovascular disorders. Variants found were annotated according to the human genome, following currently available classification used for human diseases. Sequencing from the DNA of an unrelated unaffected bonobo was also used for prediction of pathogenicity. Twenty-four variants of uncertain clinical significance (VUSs) but no pathogenic variants were found in the proband studied. Further familial, functional, and bonobo population studies are needed to determine if any of the VUSs or a combination of the VUSs found may be associated with the clinical findings. Future genotype-phenotype establishment will be beneficial for the appropriate care of the captive zoo bonobo population world-wide as well as conservation of the bobono species in its native habitat.

## Introduction

Cardiovascular disease is a leading cause of death for both human and non-human primates, who share similar genomes and many environmental and lifestyle characteristics^1^. However, while humans most often die due to coronary artery disease (CAD), non-human primates living in captivity are more often affected by hypertensive cardiomyopathy^1^. Both humans and non-human primates can also be affected by arrhythmogenic right ventricular cardiomyopathy (ARVC), a relatively uncommon autosomal dominant disease disrupting desmosomal proteins, which may lead to sudden cardiac death in young adults, or progress to heart failure later in life^2–15^.

**Figure 1:**
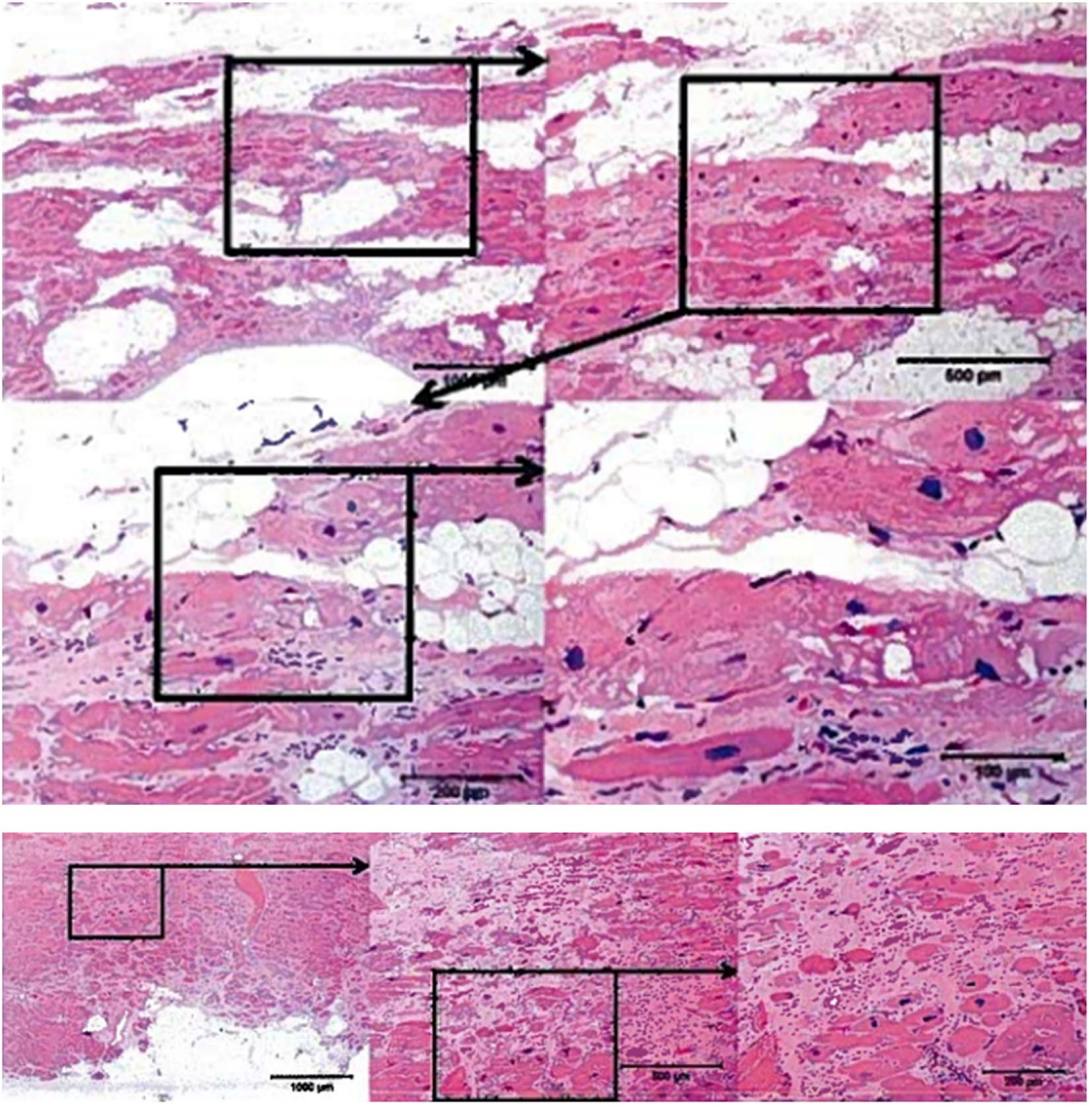
Histological analysis on myocardial specimens. Serial magnification of the same field from the necropsy specimen from the affected male bonobo demonstrating fibro-fatty replacement of right ventricle (upper row H&E 4× and 10×) and higher power images showing individual myocytes with sarcoplasmic vacuolization (H&E 20× and 40×).

In 2012, the alpha male bonobo of the Milwaukee County Zoo’s (MCZ) large troup of 20 bonobos (*Pan paniscus*) died suddenly at 38 years of age. Post-mortem examination revealed gross anatomic and fine histologic features of ARVC (Fig. 1). Since only a few thousand wild bonobos now exist in the rain forest south of the Congo River and the North American captive population of 85 bonobos are progeny of only 24 wild-caught individuals, the presence of a potentially lethal genetic variant in the small gene pool of this highly endangered species could be catastrophic for species survival. Genetic studies understanding the basis of ARVC in this restricted gene pool may also provide valuable insight into the genesis of the disease in humans and other primates, leading to risk stratification and sudden death prevention.

We have exploited the remarkable nucleotide and amino acid sequence homology between humans and bonobos to perform a candidate gene sequencing analysis of the coding regions of 10 genes previously associated with human ARVC. This approach sought to identify the possible genetic defects leading to ARVC in bonobos and to develop a test that can be used on other captive bonobos. Here, we describe the methodology used to develop our sequencing panel and report the findings in the affected bonobo but not in a healthy control individual.

## Results

We initially analyzed the coding regions and flanking exon-intron boundaries of the ARVC-associated genes *LMNA*, *CTNNA3*, *DES*, *TGFB3*, *JUP*, *TMEM43*, *PKP2*, *DSC2*, *DSG2* and *DSP* in the DNA of an MCZ’s alpha male bonobo (Lody) using a Sanger sequencing-based approach. The variants found in Lody’s DNA were compared to the human reference genome, and further annotated and analyzed for their possible involvement in the etiology of ARVC. This analysis included several *in silico* prediction algorithms as well as variant frequencies available in human population databases as described in the Methods section. Most variants, though not listed as the reference in the human genome, were listed as the reference nucleotide in the chimpanzee genome and/or the bonobo genome (https://www.ncbi.nlm.nih.gov/genome/10729), which suggested they may be tolerated and simply reflecting the difference between humans and apes. Therefore, to better assess the involvement of each variant in disease, we focused on Lody’s variants that were not found in our unaffected control bonobo, Kitty, reportedly the oldest bonobo to have lived in captivity in North America (Table 1). This resulted in the identification of two heterozygous variants of uncertain clinical significance (VUS) in the *CTNNA3* and *JUP* genes.

**Table 1:** Variants of uncertain clinical significance found in Lody (affected subject) but not in Kitty (unaffected control) by Sanger and next generation sequencing methodologies.

Gene	Coordinate (GRCh37/hg19)	Variant^†,‡,§^	ESP freq.	1000 G freq.	gnomAD freq.	Lody blood?^||^	Bonobo/Chimp reference genome?
*AGK*	chr7:141351405	c.1127 C > T (p.P376L), rs150893768	8.E-05	N/A	4.E-05	Yes	Yes/No
*APOB*	chr2:21266813	**c**.**5 A > G (p**.**D2G)**	N/A	N/A	N/A	No	NM/Yes
*CACNA1B*	chr9:141000229	c.5398 C > G (p.R1800G)	N/A	N/A	N/A	Yes	NM/No
*CACNA2D1*	chr7:81662135	**c**.**1121 A > C (p**.**N374T)**	N/A	N/A	N/A	No	Yes/Yes
*CTNNA3*	chr10:68040338	c.1774G > A (p.A592T)	N/A	N/A	N/A	yes	No/No
*DSG2*	chr18:29126381	**c**.**3032 T > C (p**.**V1011A)**	N/A	N/A	N/A	No	Yes/Yes
*FKTN*	chr9:108366531	**c**.**405 T > G (p**.**F135L)**	N/A	N/A	N/A	Yes	No/No
*FOXC2*	chr16:86601893	c.952 G > A (p.A318T)	N/A	N/A	N/A	No	NM/No
IRX4*	chr5:1878493	**c**.**1147_1149delGGT (p**.**383delT)**	N/A	N/A	N/A	Yes	No/Gap
*JUP*	chr17:39915114	c.1506 C > T (p.I502I), rs372963143	N/A	N/A	2.E-05	yes	Yes/Gap
*KCNA5*	chr12:5153465	c.152 C > A (p.P51Q)	N/A	N/A	N/A	Yes	Gap/No
*KCNH2*	chr7:150647379	c.2275 A > G (p.K759E)	N/A	N/A	N/A	Yes	No/No
*KCNH2*	chr7:150647468	c.2186 G > A (p.C729Y)	N/A	N/A	N/A	Yes	No/No
*KCNH2*	chr7:150648131	c.2023 A > G (p.T675A)	N/A	N/A	N/A	Yes	No/No
*LTBP2*	chr14:74989568	c.2584 G > A (p.V862M), rs150604905	2.E-04	N/A	1.E-04	Yes	No/No
*MYH11*	chr16:15917264	c.350 A > G (p.Y117C)	N/A	N/A	N/A	Yes	No/No
*MYH6*	chr14:23853741	c.5475 G > T (p.E1825D)	N/A	N/A	1.E-05	Yes	No/No
*MYOM1*	chr18:3215127	**c**.**95 A > C (p**.**E32A)**	N/A	N/A	N/A	Yes	No/No
*SCN5A*	chr3:38645358	c.1735G > A (p.G579R), rs199473128	N/A	N/A	8.E-05	Yes	No/No
*SLC2A10*	chr20:45354180	c.505 G > A (p.G169S), rs35151194	N/A	N/A	4.E-05	Yes	No/No
*SNTA1*	chr20:32031288	c.139 G > T (p.G47C)	N/A	N/A	N/A	Yes	Gap/No
*SYNE1*	chr6:152683327	**c**.**10298 C > T (p**.**T3433I)**	N/A	N/A	N/A	No	Yes/Yes
*SYNE1*	chr6:152683336	**c**.**10289 G > A (p**.**R3430Q)**, **rs139691734**	8.E-05	N/A	3.E-05	No	Yes/No
*TCAP*	chr17:37822316	c.458 G > A (p.R153H), rs149585781	2.E-04	N/A	2.E-04	Yes	No/No

All annotation is in relation to the human annotation for the given gene. Underlined: VUS found by Sanger sequencing method (all others by NGS). **Bold**: variants found in homozygous state (all others were heterozygous). ESP: combined allele frequency from ESP database^18^. 1000G: combined allele frequency from 1000 genomes browser^17^. gnomAD: combined allele frequency from gnomAD browser beta^20^. Search for bobono variants in its reference genome was performed using the UCSC Genome Browser blat tool on Bonobo May 2012 (Max-Planck/panPan1) Assembly (May 2016)^30^. Search for chimpanzee (chimp) variants in its reference genome was performed using the UCSC Multiz Alignments of 100 vetebrates (May 2016)^30^.

*IRX4 variant was found in heterozygosity in Kitty’s blood DNA. All other variants were not found in Kitty.

^†^Variant annotation is according to the following human transcripts: AGK NM_018238; APOB NM_000384; CACNA1B NM_000718; CACNA2D1 NM_000722; CTNNA3 NM_013266; DSG2 NM_001943; FKTN NM_006731; FOXC2 NM_005251; IRX4 NM_016358; JUP NM_002230; KCNA5 NM_002234; KCNH2 NM_000238; LTBP2 NM_000428; MYH11 NM_022844; MYH6 NM_002471; MYOM1 NM_003803; SCN5A NM_198056; SLC2A10 NM_030777; SNTA1 NM_003098; SYNE1 NM_033071; TCAP NM_003673.

^‡^All VUSs were non-synonymous variants except the *IRX4* (non-frameshift deletion), and *JUP* (synonymous).

^§^Known dbSNP rs# displayed if available^16^.

^||^All variants in this table (Table 1) were present in Lody's heart DNA.

Freq., frequency; Gap, gap in genome assembly; N/A, not applicable or not available; NM, no match to genome assembly.

**Figure 2:**
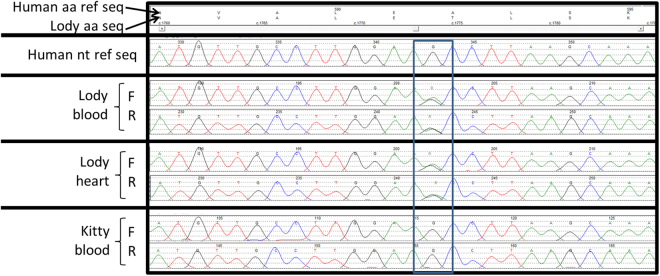
*CTNNA3* results from Sanger sequencing. Heterozygous c.1774G > A (p.A592T) variant found in Lody’s blood and heart DNA samples but not in Kitty. Rectangle encloses the variant indicated. AA, amino acid; F, forward; Nt, nucleotide; R, reverse; Ref, reference; Seq, sequence.

The first Lody-specific DNA change found was a non-synonymous heterozygous c.1774G > A (p.A592T) variant in exon 6 of the *CTNNA3* gene (Fig. 2) leading to the semi-conservative amino acid substitution of an Alanine residue with a Threonine residue at a position conserved in human, rhesus, mouse, dog, and elephant, as well as in bonobo and chimpanzee (but a Serine residue in chickens). This sequence change has not been previously described as a disease-causing variant in cardiomyopathy patients and it has not been reported in the single nucleotide polymorphism database (dbSNP), in the 2,500 subjects of the 1000 Genomes Browser, in the 6,500 subjects of the NHLBI Exome Sequencing Project (ESP) database, in the 60,312 unrelated individuals of the Exome Aggregation Consortium (ExAC), or in the gnomAD browser beta, which evaluated exome data from individuals of European, African American, Hispanic, Asian and other backgrounds, suggesting this is not a common apparently neutral variant in these populations^16–20^. Variants that affect the same amino acid p.A592G/D/S are present in the gnomAD browser beta with less than 0.00001 allele frequency^20^. While not validated for clinical use, the computer-based algorithms PolyPhen (HumDiv and HumVar) and Mutation Taster classified this variant as probably damaging or disease causing, while SIFT and SNPs&Go classified this variant as tolerated or as a neutral polymorphism.

**Figure 3:**
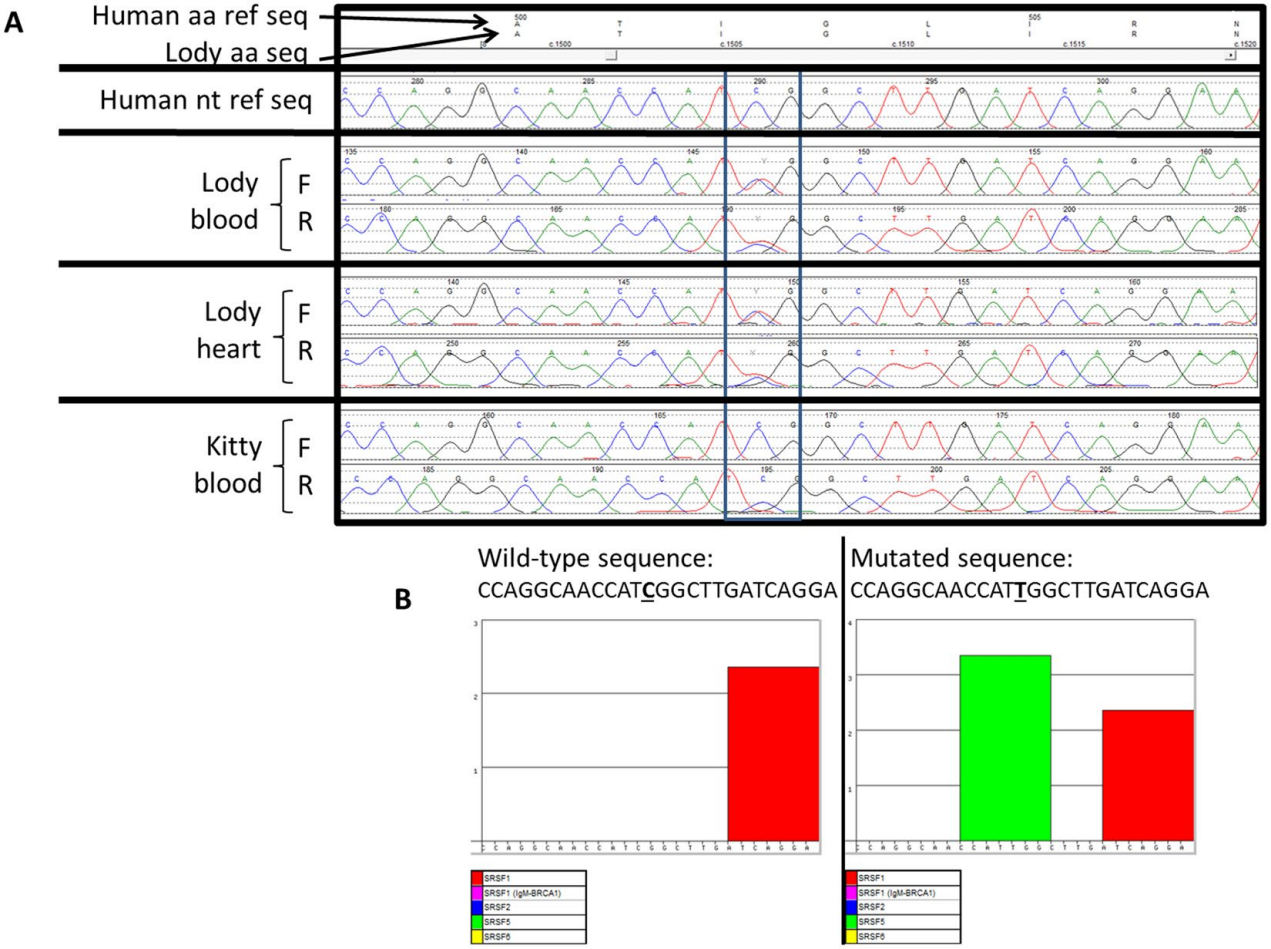
*JUP* results from Sanger sequencing and ESE prediction. (**A**) Heterozygous c.1506 C > T (p.I502I) variant found in Lody’s blood and heart DNA samples but not in Kitty. Rectangle encloses the variant indicated. (**B**) The *in silico* exonic splicing enhancer (ESE) software predicts the creation of a novel responsive sequence for the binding of the serine/arginine-rich splicing factor 5 (SRp40) in the mutated *JUP* transcript c.1506 C > T. Bold and underlined letter represents wild-type or mutated nucleotide. AA, amino acid; F, forward; Nt, nucleotide; R, reverse; Ref, reference; Seq, sequence.

The second Lody-specific variant was a synonymous heterozygous c.1506 C > T (p.I502I) variant in exon 9 of the *JUP* gene (Fig. 3A). The p.I502I is a silent variant located in the third codon of exon 9. However, the bonobo reference genome (https://www.ncbi.nlm.nih.gov/genome/10729), which is based on the sequencing of one female bonobo^21^ presents with the variant nucleotide at this position (that is, the thymine and not the cytosine, as in the human reference), while the chimpanzee reference (https://www.ncbi.nlm.nih.gov/genome/?term=pan+troglodytes) has a gap in the sequence at that position and surrounding nucleotides and thus could not be evaluated. The Isoleucine residue is conserved in human, rhesus, mouse, dog, elephant, chicken, and frog, while a similar amino acid, Valine is found in zebrafish and lamprey. This sequence change has not been previously described as a disease-causing variant in cardiomyopathy patients. This sequence change has been reported in the SNP database as rs372963143, but was not detected in the 2,500 subjects of the 1000 Genomes Browser or in the 6,500 subjects of the NHLBI ESP database, while it was found in 4/248,880 alleles (0.002%) in the gnomAD browser beta. This variant was detected in heterozygosity in 1/8806 alleles (0.01%) from African subjects, in 1/13798 alleles (0.007%) from South Asian subjects, and in 1/60274 alleles (0.002%) from European (non-Finnish) subjects, while it was not detected in all other ethnicities in the ExAC database. Overall, population data suggests this is not a common apparently neutral variant. The computer-based algorithm Mutation Taster classified this variant as disease causing, and the *in-silico* prediction tool ESE Finder predicts the addition of an SRSF5 (SRp40) putative responsive exon sequence to the mutant transcript (Fig. 3B).

Given that we did not find any pathogenic/likely pathogenic variants using our Sanger approach, we used a custom NGS panel to look for variants in the coding and splicing regions of 246 genes associated with cardiovascular disorders in Lody’s whole blood and heart and Kitty’s whole blood DNA as described in the Methods section and in a previous study^22^. In total, using the human genome GRCh37/hg19 as reference, 20,756 variants were found in Lody’s whole blood DNA, 19,817 variants were found in Lody’s formalin-fixed paraffin-embedded (FFPE) heart DNA, and 20,040 variants were found in Kitty’s whole blood DNA. There were 20,679 variants that were found in Lody’s whole blood DNA but not in Lody’s FFPE heart DNA, while there were 551 variants that were found in Lody’s FFPE heart DNA but not in Lody’s whole blood DNA (19,343 variants were found in both DNA sources and 21,230 variants were unique to one of the DNA sources). After variant classification, subtractive filtering analyses and Sanger confirmation studies, no pathogenic/likely pathogenic variants were found in Lody’s blood or heart DNA (see Methods section). However, using our NGS approach, several VUSs were found in Lody’s blood or heart DNA, and are listed in Table 1. The *CTNNA3* and *JUP* VUSs found by Sanger sequencing alone were detected by the NGS approach but did not pass our stringent post-bioinformatics filtering steps because their damage score was below 4.

In addition to sequencing approaches, we performed SNP microarray analysis of Lody’s whole blood and FFPE DNA using a microarray chip designed for the human genome. The ancestors of humans split from bonobos approximately 4 million years ago. Recent completion of the bonobo genome has revealed it to be 98.7% identical to corresponding sequences in the human genome^21^. We analyzed the data for regions of significant absence of heterozygosity (AOH), deletions or duplications involving genes that could be potentially influencing Lody’s clinical phenotype. Using the call setting described in the Methods section, we found a total of 536 calls in the heart and 907 calls in the blood. After further data curation to exclude AOH, deletion and duplication calls with no OMIM genes, we found a total of 242 calls in the heart (16 AOH, 73 gain, and 153 loss) and 479 calls in the blood (3 AOH, 5 gain, and 471 loss) (Table 2 and Supporting Information Table S1). There were no significant calls involving the 10 ARVC genes sequenced by Sanger methods. Additionally, we determined if there were patterns of long stretches with AOH, also termed runs of homozygosity (ROH). ROH has been utilized to determine the level of parental relatedness in an individual^23–25^. We did not observe substantial ROH in Lody, suggesting there has not been inbreeding in immediate generations. Overall, data for the heart FFPE sample was suboptimal, likely due to the poor quality of the DNA for use in microarrays.

**Table 2:** Summary of SNP microarray analysis calls for Lody (affected subject).

Lody DNA	All SNP calls				Calls with ≥ 1 genes				Calls with ≥ 1 OMIM genes			
	AOH	Gain	Loss	Total	AOH	Gain	Loss	Total	AOH	Gain	Loss	Total
Heart (FFPE)	16	92	428	536	16	81	223	320	16	73	153	242
Whole blood	3	10	894	907	3	5	621	629	3	5	471	479

AOH, absence of heterozygosity; FFPE, formalin-fixed paraffin-embedded.

## Discussion

The primary goal of our study was to investigate the molecular basis of the seeming ARVC in a specific subject, a 38-year-old founder bonobo (Lody), who died suddenly of a histologically characterized arrhythmogenic cardiomyopathy. A definitive identification of the molecular culprit(s) would have aided in identifying individuals harboring the genetic variant(s) and attaining proper risk-stratification of Lody’s offspring, thus warranting a rigorous surveillance and appropriate medical treatment. Two of three full-sibling descendants from this founder’s mating with a founder female with progressive limb weakness of unknown etiology had early deaths, further supporting the potential inherited nature of the disease, thus adversely affecting future generations and the sustainability of this species. A secondary goal of our project was to find variants that would shed new light into the genetics of heart diseases in bonobos and develop a test panel that could be utilized by other institutions and provide a replicable, non-invasive test for all great apes to be easily screened for cardiovascular disease. This preliminary information may help identify and diagnose individuals at an earlier stage of disease progression and thus enroll them in training to participate in echocardiogram tests and other non-invasive monitoring and treatment plans. Early diagnosis is vitally important to prevention and treatment, and is especially important for the bonobo species, which is currently listed as endangered in their wild habitat (west-central Africa). Finally, in the case of the bonobo, these results will assist the Bonobo SSP in making more knowledgeable breeding recommendations for the already limited captive population.

In our efforts to identify the genetic etiology for the clinical presentation for Lody, we sequenced the coding regions and flanking exon-intron boundaries of 10 ARVC-associated genes (*LMNA*, *CTNNA3*, *DES*, *TGFB3*, *JUP*, *TMEM43*, *PKP2*, *DSC2*, *DSG2*, and *DSP*) in our subject and compared his data to that of an unaffected female bonobo (Kitty). The initial method of choice for this proposal was Sanger sequencing as it provided the flexibility necessary to design the assay, the complete coverage of the targeted regions, and avoidance of duplicated homologous loci. We did not find any variants with sufficient evidence to be clearly pathogenic in the blood or heart tissue from Lody; however, we found two VUSs in the *CTNNA3* and *JUP* genes that were found in Lody’s whole blood and heart DNA, but not in Kitty’s whole blood DNA. We do not have enough functional and segregation data to support that any of these variants may alone explain Lody’s phenotype, but we cannot rule out that one of them or the combination of these two variants, may be related to the etiology of ARVC in our subject.

The catenin (cadherin-associated protein), alpha 3 (CTNNA3) protein is a member of the vinculin/alpha-catenin family and has a role in cell-cell adhesion in muscle cells, with mutations being associated with ARVC^26^. The heterozygous c.1774G > A (p.A592T) variant found in Lody has not been studied functionally, has not been previously described in cardiomyopathy human patients or control populations, and *in silico* data for prediction of effect on the structure/function of the protein is inconclusive. Two rare (<0.05% in the ExAC database) missense variants are found in the same residue, p.A592D and p.A592G (rs768797369), but no functional or segregation data is available for these variants. The functional significance of this sequence change is not known at present and its contribution to Lody’s disease phenotype cannot definitely be determined.

The junction plakoglobin (JUP) protein is a member of the catenin family and is present in desmosomes and intermediate junctions, with mutations being associated with ARVC^27^. The silent heterozygous c.1506 C > T (p.I502I) variant found in Lody has not been studied functionally, has not been previously described in cardiomyopathy human patients, and is very rare in control populations. The *in silico* exonic splicing enhancer (ESE) software predicts the creation of a novel responsive sequence for the binding of the serine/arginine-rich splicing factor 5 (SRp40) in the mutated transcript. However, we cannot determine how this may affect the structure/function of the protein, and thus, we cannot determine its contribution to Lody’s phenotype. Interestingly, the bonobo reference genome, which is based on the DNA sequence of one female individual^21^, also presents with the c.1506 T. Currently, clinical information about this individual is limited but indicates that she is healthy and is approximately 22.5 years old (personal communication with Jean-Pascal Guery, La Vallee des Singes zoological director, 04/20/2016). At present, we cannot absolutely assure that the presence of the thymine at this position is benign.

Given the Sanger sequencing results, we performed supplementary sequencing using NGS for a group of 246 cardiovascular genes, but did not find any pathogenic/likely pathogenic variants. Instead, we found several additional VUSs in Lody’s blood and/or heart DNA which may be causing or contributing to his clinical phenotype alone or in combination with other variants. Finally, we found no significant deletions, duplications, or AOH in the blood or heart tissue from Lody using chromosomal microarray analysis testing.

We acknowledge several limitations to our study. Further studies would be desirable to better understand the functional effect of each variant found in this report. Our results are based on the analysis of one affected and one unaffected individuals, which is not powerful enough to resolve the potential pathogenicity of the variants we found, since they are not highly characterized variants. Also, it is possible that some of the variants were overlooked because they were found in both subjects, and we should consider the possibility of low penetrance of such variants and/or the effect of these variants on disease expressivity. In addition, our population data is based on human databases such as the gnomAD, ExAC browser, the ESP, and the 1000 genomes project, which, although very helpful, are not necessarily optimal for the bonobo interpretation. Furthermore, the current bonobo reference genome is based on the sequencing of the genome of a single female, Ulindi. Personal communication with La Vallee des Singes, the current housing institution for Ulindi, reported that she was born on August 1993, was strong and healthy at the age of 22 years (when communication was established), and had a healthy one-year-old baby. Finally, although NGS resequencing is employed daily in clinical practice for human subjects, the application of resequencing for bonobo is limited with the current UCSC Genome Browser assembly ID: panPan1 uploaded in 2012. Although, *de novo* genome sequencing is possible, it requires an enormous bioinformatics and post-bioinformatics effort for variant calling and curation, which is beyond the scope of this current project.

To our knowledge, this is the first comprehensive genetic approach using targeted gene sequencing, next generation sequencing, and genome-based chromosomal microarray analysis attempting to find a genetic etiology for the ARVC presentation of a deceased alpha male bonobo from the MCZ by searching for pathogenic variants in genes previously linked to ARVC, as well as in 246 cardiovascular genes. The sequencing of other affected relatives and segregation analyses may further support the pathogenic role for each variant or support the multi-locus additive effect of a combination of variants if they co-segregate with the disease phenotype in Lody’s family members. Alternatively, the sequencing of the genes where VUSs were found or whole exome sequencing in several additional affected and unaffected captive bonobos, as well as from all four non-human primate genera may be helpful to elucidate the basis of such potentially devastating disease. We hope that through the development of this cardiovascular assessment strategy, the bonobo, as well as the other great apes, will benefit from the molecular technologies currently available and therefore be more likely to live a longer, more productive life, and be able to contribute to the breeding and preservation of their species. We have a rare opportunity to utilize the large, collaborative efforts and data banks from human studies for the benefit of our closely-related apes. Finally, findings from non-human primates may make clinical significant contributions to the overall understanding of ARVC in human subjects.

## Methods

### Study Subjects

“Lody,” the founder male of the MCZ bonobo troop, died of sudden cardiac arrest at the age of 39 after several years suffering from heart failure with severely reduced ejection fraction (EF = 21%) and arrhythmias, despite medical therapy employing ACE inhibitors, beta blockers, and low dose aspirin. The autopsy revealed no evidence of gross atherosclerosis and normal cardiac chambers dimensions. In addition to the more typical left ventricular myocardial fibrosis, gross anatomical and fine histologic analysis revealed unexpected findings of right ventricular fibro-fatty replacement of the myocardium and trabeculated endocardial surfaces in both ventricles. The right ventricular focal transmural fatty replacement of the myocardium along with patchy subendocardial fibrosis in the left ventricular free wall was histologically identical to ARVC in humans (Fig. 1).

“Kitty,” an unrelated female bonobo who died at age 64 years with normal cardiac findings at necropsy, served as an unaffected control for gene sequencing. All post-mortem studies were approved by the Research Committee of the MCZ in accordance with principles of the Bonobo Species Survival Project of the Association of Zoos and Aquariums. Imaging and other phenotypic analyses including MRIs, Holter monitoring, and ECGs often used in human patients are not available for the study subjects, which were wild animals and not domesticated; and these and similar diagnostic procedures require general anaesthesia to be performed in bonobos, even those in captivity.

### DNA Extraction

Lody’s frozen whole blood collected in a purple-top tube, Lody’s FFPE) heart tissue, and Kitty’s frozen whole blood collected in a purple-top tube at the Milwaukee County Zoo were sent to the Indiana University School of Medicine Molecular Genetics Diagnostic Laboratory of the Department of Medical and Molecular Genetics, Indiana University School of Medicine, for DNA extraction and sequence analysis. DNA from Lody and Kitty’s whole blood were extracted using Qiagen’s Gentra Puregene Blood Kit (Qiagen, Germantown, MD) following the manufacturer’s instructions. DNA from Lody’s FFPE heart tissue was extracted using Qiagen’s All Prep DNA/RNA FFPE Kit following the manufacturer’s instructions.

### Design of PCR Primers

Primers were designed using Primer3 web^28^ for the coding regions and flanking exon-intron boundaries of the *LMNA*, *CTNNA3*, *DES*, *TGFB3*, *JUP*, *TMEM43*, *PKP2*, *DSC2*, *DSG2*, and *DSP* genes using the corresponding human genes from assembly GRCh37/hg19 as templates^29^. At the time this project started, the first assembly of the bonobo genome (panpan1) was recently published^21^ (https://www.ncbi.nlm.nih.gov/genome/10729) and was available for BLAST search to identify sequence homology between the more common *Pan troglodytes* (https://www.ncbi.nlm.nih.gov/genome/?term=pan+troglodytes; chimpanzee, the chimpanzee and bonobo genomes share 99.6% identity)^21^ and *Homo sapiens*. However, the major genome browsers^29–31^ did not support the genome analysis for bonobo and a refined annotation was not available at that time. Primers were verified using *in silico* PCR^30^ using the chimpanzee genome to ensure primers would align properly. The UCSC Browser BLAT tool for chimpanzee genome was used to ensure that a single product would result^30^. The NCBI Blast tool for *P*. *paniscus* was used to check for regions containing SNPs between chimpanzee and bonobo (to avoid any possible allele dropout)^32^.

### PCR, BigDye Sequencing, and Analysis of Variants

Genomic DNA from Lody (whole blood and heart tissue) and from Kitty (whole blood) was amplified by PCR using Qiagen’s HotstarTaq DNA Polymerase® and lab designed primers from Integrated DNA Technologies (IDT, Coralville, IA) with M13 tails used for BigDye (Sanger) sequencing (Supporting Information Table S2). A 25 µL reaction was set up containing 40 ng of DNA, 1 µM of primer pairs, 2 mM of dNTPs, 2.5 µL 10× buffer, 5 µL 5× Q-solution, and 1 µL 25 mM MgCl^2+^. Touchdown PCR was performed using the following conditions: 95 °C for 15 min; 14 cycles of 94 °C for 45 sec, 30 sec at 65 °C with a −0.5 °C change per cycle and 1 min at 72 °C; 24 cycles of 94 °C for 45 sec, 58 °C for 30 sec and 1 min at 72 °C; 72 °C for 10 min. Small aliquots of the PCR products were run by electrophoresis on a 1% agarose gel with ethidium bromide to confirm appropriate amplification. Products were then sequenced using an Applied Biosystems 3130xl or 3500xl Genetic Analyzer in conjunction with the ABI BigDye® Terminator v3.1 Cycle Sequencing kit chemistry and protocol (ABI, Foster City, CA). Sequences where aligned to each gene and analyzed using Mutation Surveyor software V4.0.7 (SoftGenetics, State College, PA).

Variants found in Lody were annotated according to the human genome assembly GRCh37/hg19 using the following transcripts: *LMNA* (NM_170707), *CTNNA3* (NM_013266), *DES* (NM_001927), *TGFB3* (NM_003239), *JUP* (NM_002230), *TMEM43* (NM_024334), *PKP2* (NM_004572), *DSC2* (NM_024422), *DSG2* (NM_001943), and *DSP* (NM_004415). Variants found in Lody were characterized based on mutation type, location, and zygosity. Presence of the variant in the bonobo genome from the NCBI Blast tool for *P*. *paniscus* or Blat tool from UCSC Genome Browser on Bonobo May 2012 (Max-Planck/panPan1) Assembly (May 2016) and in the chimpanzee genome from UCSC browser reference, existence of a corresponding human SNP from the dbSNP, the minor allele frequency for the SNP from the 1000 Genomes browser, and variant presence in the NHLBI Exome Sequencing Project, and in the Exome Aggregation Consortium (ExAC), Cambridge, MA were used to determine how common each variant was in the human populations studied^16–19,30,32^. Furthermore, variant pathogenicity was further determined based on existing literature cited in the Human Gene Mutation Database (HGMD), on GERP and Grantham scores listed in the ESP database, and other *in silico* prediction analyses including PolyPhen, SIFT, Mutation Taster, SNPs&Go, Berkeley Drosophila Genome Project, ESE Finder, and miRBase^33–44^. Finally, variants were determined to have more burden if they were found in Lody (our subject) but not in Kitty (our control), as those in common between the two individuals were judged to be either unlikely to be clinically relevant or to be of low penetrance (see Table 1).

### Next Generation Sequencing and Analysis of Variants

A previously described custom next generation sequencing (NGS) panel containing probes for human DNA was used in the DNA from Lody’s whole blood and heart and from Kitty’s blood to sequence the coding and splicing regions of 246 genes associated with cardiovascular disorders including arrhythmias, cardiomyopathies, congenital heart defects, aortopathy, connective tissue disorders, Noonan spectrum disorders, pulmonary arterial hypertension, metabolic disorders that afflict the heart and lipid disorders^22^. Briefly, paired-end sequencing was performed using an Illumina MiSeq sequencer (Illumina, Inc., San Diego, CA), followed by read alignment using the BWA software, local realignment, base quality recalibration, and variant identification using the GATK software, and variant annotation using ANNOVAR^22^. A 300× average depth of coverage was obtained among variants for the three samples.

Variants found in Lody’s whole blood were divided into those found in the HGMD and those not found in the HGMD (non-HGMD). The HGMD variants were classified as being pathogenic/likely pathogenic, pathogenic/likely pathogenic in autosomal recessive disorders, modifier, VUS, or benign/likely benign based on our interpretation from the literature^22^. In order to filter the non-HGMD variants (variants that did not have a previous association with human diseases) and find those with higher likelihood of having disease association, we selected variants that were in coding regions, excluded synonymous and nonsynonymous that were found in more than 2% of the populations in the 1000 Genomes browser, the NHLBI Exome Sequencing Project, and the gnomAD browser beta^20^, excluded variants that had a damage score lower than 4 (based on software *in silico* prediction of damage to protein structure/function)^22^, excluded synonymous and non-frameshift *TTN* variants, excluded variants that mapped to regions of known segmental duplications, and excluded non-frameshift variants that were found in more than 2% of the populations in the 1000 Genomes browser, in the NHLBI Exome Sequencing Project, or in the gnomAD browser beta. Following, a subtractive analysis was performed to select only those resulting HGMD and non-HGMD variants of acceptable sequencing quality scores that were specific to Lody (not in Kitty, unless heterozygous in Kitty for a homozygous variant in Lody), as those in common between the two individuals were judged to be either unlikely to be clinically relevant or to be of low penetrance. Finally, the presence of each variant in the bonobo and in the chimpanzee genome was annotated as explained above. This analysis resulted in seral VUSs but no pathogenic/likely pathogenic variants. VUSs found to have acceptable quality and sequence depth scores were carefully chosen (Table 1).

Variants found in Lody’s FFPE heart but not in Lody’s blood extracted DNA were divided into those found in the HGMD and those not found in the HGMD (non-HGMD). Filtering and classification of variants was performed as explained above. This analysis resulted in seral VUSs but no pathogenic/likely pathogenic variants. VUSs found to have acceptable quality and sequence depth scores were carefully chosen (Table 1).

### SNP Microarray Analysis

Single nucleotide polymorphism (SNP) array analysis was performed on Lody’s blood and FFPE heart DNA using the AffymetrixCytoScan HD array that contains 1,953,246 human non-polymorphic markers and 743,304 human SNP markers (Affymetrix, Santa Clara, CA). Allele peaks and signal intensity log2 ratios were analyzed with the Chromosome Analysis Suite (ChaS) software (Version 2.0.0.195) (Affymetrix) to determine gains, losses, and copy-neutral absence of heterozygosity (CN-AOH). The settings used for calls were as follows: 60 marker counts, 300 kbp for gain; 60 marker counts, 300 kbp for loss; 3000 kbp for AOH. Data was analyzed based on the NCBI human genome build GRCh37/hg19.

## Electronic supplementary material

Supplementary Tables

## References

[1] Lowenstine LJ, McManamon R, Terio KA (2016). Comparative pathology of aging great apes: bonobos, chimpanzees, gorillas, and orangutans. Vet. Pathol..

[2] Lammey ML, Lee DR, Ely JJ, Sleeper MM (2008). Sudden cardiac death in 13 captive chimpanzees (Pan troglodytes). J. Med. Primatol..

[3] Corely KC, Shiel FO, Mauck HP, Clark LS, Barber JH (1977). Myocardial degeneration and cardiac arrest in squirrel monkey: physiological and psychological correlates. Psychophysiology.

[4] Doane CJ, Lee DR, Sleeper MM (2006). Electrocardiogram abnormalities in captive chimpanzees (Pan troglodytes). Comp. Med..

[5] Hollander W, Prusty S, Kirkpatrick B, Paddock J, Nagraj S (1977). Role of hypertension in ischemic heart disease and cerebral vascular disease in cynomolgus monkey with coarctation of the aorta. Circ. Res..

[6] Munson L, Montali RJ (1990). Pathology and disease of great apes at the National Zoological Park. Zoo Biol..

[7] Myerburg, R. J., Interian, A., Simmons, J. & Castellanos, A. Sudden cardiac death. (ed. Zipes, D. P.) Cardiac Electrophysiology: From Cell to Bedside 720–731 (WB Saunders, 2004).

[8] Schulman FY, Farb A, Virmani R, Montali RJ (1995). Fibrosing cardiomyopathy in captive Western Lowland gorillas (Gorilla, gorilla, gorilla) in the United Sates: A retrospective study. J. Zoo. Wildlife Med..

[9] Seiler BM (2009). Spontaneous heart disease in the adult chimpanzee (Pan troglodytes). J. Med. Primatol..

[10] Center for Disease Control and Prevention, National Center for Health Statistics. Summary Health Statistics for U.S. Adults: national health interview survey, 2003. *Vital Health Stat*. **10(225)** Ser (2005). 25116227

[11] Hansen JF, Alford PL, Keeling ME (1984). Diffuse myocardial fibrosis and congestive heart failure in an adult male chimpanzee. Vet. Pathol..

[12] Hubbard GB, Lee DR, Eichberg JW (1991). Diseases and pathology of chimpanzees at the Southwest Foundation for Biomedical Research. Am. J. Primatol..

[13] McNamara, T., Dolensek, E. P., Lui, S.-K. & Dierenfeld, E. Cardiomyopathy associated with vitamin E deficiency in two mountain lowland gorillas. *Proceedings of the First International Conference on Zoological and Avian Medicine*; East Northport, NY: AAV/AAZV p.493 (1987).

[14] Schmidt RE (1978). Systematic pathology of chimpanzees. J. Med. Primatol..

[15] World Health Organization. World health statistics 2006. Geneva: World Health Organization (2006).

[16] dbSNP. Available at: http://www.ncbi.nlm.nih.gov/SNP/ (Accessed: 16^th^ January 2017).

[17] 1000genomes browser. Available at http://www.ncbi.nlm.nih.gov/variation/tools/1000genomes/ (Accessed: 16^th^ January 2017).

[18] NHLBI Exome Sequencing Project (ESP). Available at http://evs.gs.washington.edu/EVS/ (Accessed: 16^th^ January 2017).

[19] Exome Aggregation Consortium (ExAC), Cambridge, MA. Available at http://exac.broadinstitute.org (Accessed: 16^th^ January 2017).

[20] gnomAD browser beta. Available at http://gnomad.broadinstitute.org/ (Accessed: 16^th^ January 2017).

[21] Prüfer K (2012). The bonobo genome compared with the chimpanzee and human genomes. Nature..

[22] Celestino-Soper PB (2015). Evaluation of the genetic basis of familial aggregation of pacemaker implantation by a large next generation sequencing panel. PLoS One..

[23] Prado-Martinez J (2013). Great ape genetic diversity and population history. Nature..

[24] Pemberton TJ (2012). Genomic patterns of homozygosity in worldwide human populations. Am. J. Hum. Genet..

[25] McQuillan R (2008). Runs of homozygosity in European populations. Am. J. Hum. Genet..

[26] CTNNA3 [OMIM 607667]. Available at https://www.ncbi.nlm.nih.gov/omim/?term=607667 (Accessed: 16^th^ January 2017).

[27] JUP [OMIM 173325]. Available at https://www.ncbi.nlm.nih.gov/omim/?term=173325 (Accessed: 1^6t^h January 2017).

[28] Primer3web. Available at http://primer3.wi.mit.edu/ (Accessed: 16^th^ January 2017).

[29] Ensembl. Available at http://www.ensembl.org (Accessed: 16^th^ January 2017).

[30] University of California Santa Cruz (UCSC) Genome Browser. Available at http://genome.ucsc.edu/ (Accessed: 1^6t^h January 2017).

[31] National Center for Biotechnology Information (NCBI). Available at https://www.ncbi.nlm.nih.gov/ (Accessed: 16^th^ January 2017).

[32] National Center for Biotechnology Information (NCBI) Blast tool for *P*. *paniscus*. Available at http://www.ncbi.nlm.nih.gov/genomes/geblast.cgi?taxid=9597 (Accessed: 16^th^ January 2017).

[33] Human Gene Mutation Database. Available at https://portal.biobase-international.com/cgi-bin/portal/login.cgi (Accessed: 16^th^ January 2017).

[34] Stenson PD (2003). Human Gene Mutation Database (HGMD): 2003 update. Human mutation.

[35] Stenson, P. D. *et al*. The Human Gene Mutation Database (HGMD) and its exploitation in the fields of personalized genomics and molecular evolution. *Curr*. *Protoc*. *Bioinformatics* Chapter Unit1.13. 1010020471250953bi0113s39 (2012). 10.1002/0471250953.bi0113s39 22948725

[36] Cooper, D. N., Stenson, P. D. & Chuzhanova, N.A. The Human Gene MutationDatabase (HGMD) and its exploitation in the study of mutational mechanisms. *Curr*. *Protoc*. *Bioinformatics* Chapter 1: p. Unit1.13. 10.1002/0471250953.bi0113s12 (2006). 10.1002/0471250953.bi0113s12 18428754

[37] PolyPhen. Available at http://genetics.bwh.harvard.edu/pph2/ (Accessed: 16^th^ January 2017).

[38] SIFT. Available at http://sift.jcvi.org/www/SIFT_seq_submit2 html. (Accessed: 1^6t^h January 2017).

[39] Mutation Taster. Available at http://www.mutationtaster.org/ (Accessed: 16^th^ January 2017).

[40] SNPs&Go. Available at http://snps-and-go.biocomp.unibo.it/snps-and-go/ (Accessed: 16^th^ January 2017).

[41] Berkeley Drosophila Genome Project. Available at http://www.fruitfly.org/seq_tools/splice.html (Accessed: 16^th^ January 2017).

[42] Smith PJ (2006). An increased specificity score matrix for the prediction of SF2/ASF-specific exonic splicing enhancers. Hum. Mol. Genet..

[43] Cartegni L, Wang J, Zhu Z, Zhang MQ, Krainer AR (2003). ESEfinder: a web resource to identify exonic splicing enhancers. Nucleic Acids Res..

[44] miRBase. Available at http://www.mirbase.org/search.shtml (Accessed: 16^th^ January 2017).

